# Translating the role of PARP inhibitors in triple-negative breast cancer

**DOI:** 10.18632/oncoscience.474

**Published:** 2019-01-31

**Authors:** Michèle Beniey, Takrima Haque, Saima Hassan

**Affiliations:** Division of Surgical Oncology, Department of Surgery, Centre Hospitalier de l'Université de Montréal (CHUM); Université de Montréal; Institut du cancer de Montréal and Centre de Recherche du CHUM (CRCHUM), Montreal, Quebec, Canada

**Keywords:** triple-negative breast cancer, PARP inhibitors, predictors of response

Triple-negative breast cancer (TNBC) is a heterogeneous and aggressive subtype of breast cancer that lacks expression of estrogen receptor (ER), progesterone receptor (PR), and HER2. Patients with TNBC have a poorer overall survival and tend to progress within three years of diagnosis [1]. The therapeutic management of TNBC is a significant challenge, since patients cannot benefit from antihormonal or HER2-directed therapy, and are commonly offered several types of cytotoxic chemotherapy.

A family of targeted therapeutic agents that have recently been approved by the FDA for 10-15% of TNBC patients with germline mutations in BRCA1/2 are Poly (ADP-Ribose) Polymerase inhibitors (PARPi) [2]. PARPi target the DNA repair process and have two main mechanisms of action: synthetic lethality, whereby the double-hit of PARP1/2 and BRCA1/2 renders homologous recombination dysfunctional; and PARP-DNA trapping, which creates cytotoxic “poisons” that result in cell death [1]. As single agents, the efficacy of two PARPi, olaparib and talazoparib, was demonstrated in two independent phase 3 randomized controlled trials in metastatic HER2-negative BRCA1/2-mutated patients who received less than three lines of chemotherapy. These trials demonstrated comparable results, where the PARP inhibitor was associated with an improvement in progression-free survival of about three months and an objective response rate near 60%, in comparison to physician's choice of single-agent chemotherapy [2].

While these results are very encouraging for patients with BRCA1/2 mutations, it is still not well understood if we can broaden the use of PARP inhibitors to other TNBC patients. Clinical interest in TNBC is well-founded since these tumors have been shown to demonstrate BRCAness, sharing many clinicopathological features as BRCA-mutant tumors, and are associated with defective DNA repair mechanisms [1]. A few clinical trials have addressed the role of PARPi in TNBC patients, and the results are difficult to interpret. Olaparib was evaluated in a cohort of twenty-six breast cancer patients, who were heavily pretreated with up to seven lines of chemotherapy, and no objective response was observed in BRCA-mutant or BRCA wild-type patients [3]. Veliparib was tested in combination with four therapeutic agents, and there was no added benefit of this PARPi [2]. In combination, the optimal dosing required for efficacy and minimal toxicity of PARPi has not been well understood, and the optimal sequencing strategy of PARPi with chemotherapy is yet to be reported. However, there is strong evidence from preclinical studies that support the use of PARPi in BRCA wild-type breast cancer. We and others have reported sensitivity to olaparib, independent of BRCA-mutation status in a panel of breast cancer cell lines [4-6]. BRCA wild-type tumors were also found to be sensitive to olaparib and talazoparib *in-vivo*, in two distinct cohorts of breast cancer patient-derived xenografts [7, 8].

**Figure 1:**
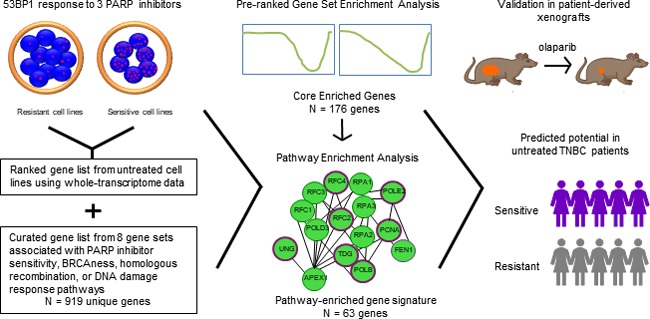
Schematic representation of the derivation of our 63-gene signature associated with response to PARP inhibition

It is plausible that we will observe greater efficacy of PARP inhibitors amongst a subpopulation of TNBC patients. Various approaches have been used to identify genetic determinants of response to PARP inhibition or BRCAness including siRNA/shRNA knockdown libraries or computational methods in conjunction with *in-vitro* responses. We used a novel approach to predict response to PARPi by defining sensitivity and resistance using the DNA damage response and identifying gene predictors with gene set and pathway enrichment analysis (Figure 1) [6]. From a collection of TNBC cell lines, we evaluated the efficacy of three PARP inhibitors: veliparib, olaparib, and talazoparib, using a 10-day chemosensitivity assay [6]. We derived IC50 values using an automated cell-count approach, and quantified the DNA damage response by enumerating 53BP1 foci with high-content imaging and single-cell analysis. We calculated EC50 values for the mean number of 53BP1 foci per cell, and percentage of cells positive for 53BP1. We found a strong correlation between IC50 values and 53BP1 EC50 values.

We then used the 53BP1 response to categorize the cell lines into sensitive and resistant groups to create a gene rank list using whole-transcriptome data of these cell lines prior to treatment. We then generated a curated database of eight gene sets previously found to be associated with either response to olaparib, talazoparib, BRCA1/2 mutation status, or BRCAness, and performed a pre-ranked gene set enrichment analysis. We used the identified core-enriched genes to undertake a pathway enrichment analysis to determine the statistically significant involved pathways, from which we created our unique 63-gene signature. We validated our gene signature in a cohort of seven patient-derived xenografts treated with olaparib. Our 63-gene signature demonstrated an overall accuracy of 86%, taking into account sensitivity and specificity, and outperformed seven other gene sets associated with response to PARPi or BRCAness. Furthermore, we found that our 63-gene signature predicted sensitivity to PARPi in 45% of untreated TNBC patients [6].

In summary, PARPi have made great progress clinically, now becoming part of the therapeutic armamentarium for BRCA-mutant TNBC patients. We now need to better understand the role of PARPi in BRCA wild-type TNBC patients. We have derived a 63-gene signature to better identify which TNBC tumors would benefit from PARPi. Work is ongoing to further validate our gene signature in a larger cohort of PDXs and to determine its prevalence in different clinical settings. Ultimately, we will need to validate the predictive potential of our 63-gene signature within the context of a clinical trial, to determine if a subset of TNBC patients will benefit from PARP inhibition, with the overarching aim to improve their prognosis.
